# Expression and activity of angiotensin-regulating enzymes is associated with prognostic outcome in clear cell renal cell carcinoma patients

**DOI:** 10.1371/journal.pone.0181711

**Published:** 2017-08-15

**Authors:** Peio Errarte, Maider Beitia, Itxaro Perez, Lorea Manterola, Charles H. Lawrie, Jon Danel Solano-Iturri, Julio Calvete-Candenas, Miguel Unda, José I. López, Gorka Larrinaga

**Affiliations:** 1 Department of Nursing I, Medicine and Nursing Faculty, University of the Basque Country (UPV/EHU), Leioa, Bizkaia, Spain; 2 Department of Physiology, Medicine and Nursing Faculty, University of the Basque Country (UPV/EHU), Leioa, Bizkaia, Spain; 3 BioCruces Research Institute, Barakaldo, Bizkaia, Spain; 4 Molecular Oncology, Biodonostia Research Institute, Donostia, Gipuzkoa, Spain; 5 Ikerbasque, Basque Foundation for Science, Bilbao, Spain; 6 Radcliffe Department of Medicine, University of Oxford, Oxford, United Kingdom; 7 Department of Pathology, Basurto University Hospital, Bilbao, Bizkaia, Spain; 8 Deparment of Clinical Oncology, Virgen de la Macarena University Hospital, Sevilla, Spain; 9 Department of Urology, Basurto University Hospital, Bilbao, Bizkaia, Spain; 10 Department of Pathology, Cruces University Hospital, University of the Basque Country (UPV/EHU), Barakaldo, Bizkaia, Spain; National Institute of Health, UNITED STATES

## Abstract

The discovery of the intrarenal renin-angiotensin system (iRAS), which regulates angiogenesis, cell differentiation and proliferation, has opened new perspectives in the knowledge of kidney carcinogenesis. In this study we analyzed the immunohistochemical expression and fluorimetric activity of four key peptidases of iRAS in tumor tissue (n = 144) and serum samples (n = 128) from patients with renal neoplasms. Neutral endopeptidase (NEP/CD10), Angiotensin-converting enzyme-2 (ACE2), and aminopeptidase A (APA) were expressed in tumor cells whilst Angiotensin-converting enzyme (ACE) was expressed in the endothelial cells of intratumor blood vessels. The expression of ACE, ACE2 and NEP/CD10 was highest in clear cell renal cell carcinoma (CCRCC) and papillary renal cell carcinoma (PRCC). The expression of these enzymes correlated with CCRCC aggressiveness. In addition, NEP/CD10 correlated with 15-year overall survival. On the other hand, APA expression was decreased in CCRCC with higher grade and stage. The loss of expression of APA independently correlated with a worse 15-year overall survival. Serum activity of ACE2, NEP/CD10 and APA was significantly higher in renal tumor patients than in healthy subjects. Serum ACE activity was lower in high grade and metastatic CCRCC patients, and NEP/CD10 activity was negatively correlated with UISS (UCLA Integrated Staging System) and SSIGN (Mayo Clinic stage, size, grade and necrosis model) scores and with overall survival of CCRCC patients. These results suggest a metabolic imbalance of iRAS in renal tumors. This finding should be taken into account in the search of new diagnostic, prognostic and therapeutic tools for this disease.

## Introduction

Renal cell carcinoma (RCC) is one of the top-ten most frequent malignancies in Western Countries. Moreover, epidemiological data reveal that its incidence has been steadily increasing in Europe and United States during the last years [1,2]. Clear cell renal cell carcinoma (CCRCC) is by far the most frequent histological subtype, accounting for approximately 75–80% of the cases, followed by papillary renal cell carcinoma (PRCC) (10–15%) [3]. Both types are proposed to originate from the proximal convoluted tubule [4]. Chromophobe renal cell carcinoma (ChRCC) and renal oncocytoma (RO), both originating from the intercalated cells of the distal nephron, are thought to share a common lineage and account for approximately 5% of the cases each [3,4].

RCC represents a major health problem primarily due to its resistance to current chemo- and radiotherapy protocols [3,5]. Presently, there are no clinical markers able to detect these diseases whilst asymptomatic and potentially curable. Only classic pathological parameters such as histological subtype, tumor stage and grade may be of help. However, these parameters give incomplete information since a significant percentage of renal tumors follow an unpredictable clinical course nowadays [3,6].

**Fig 1:**
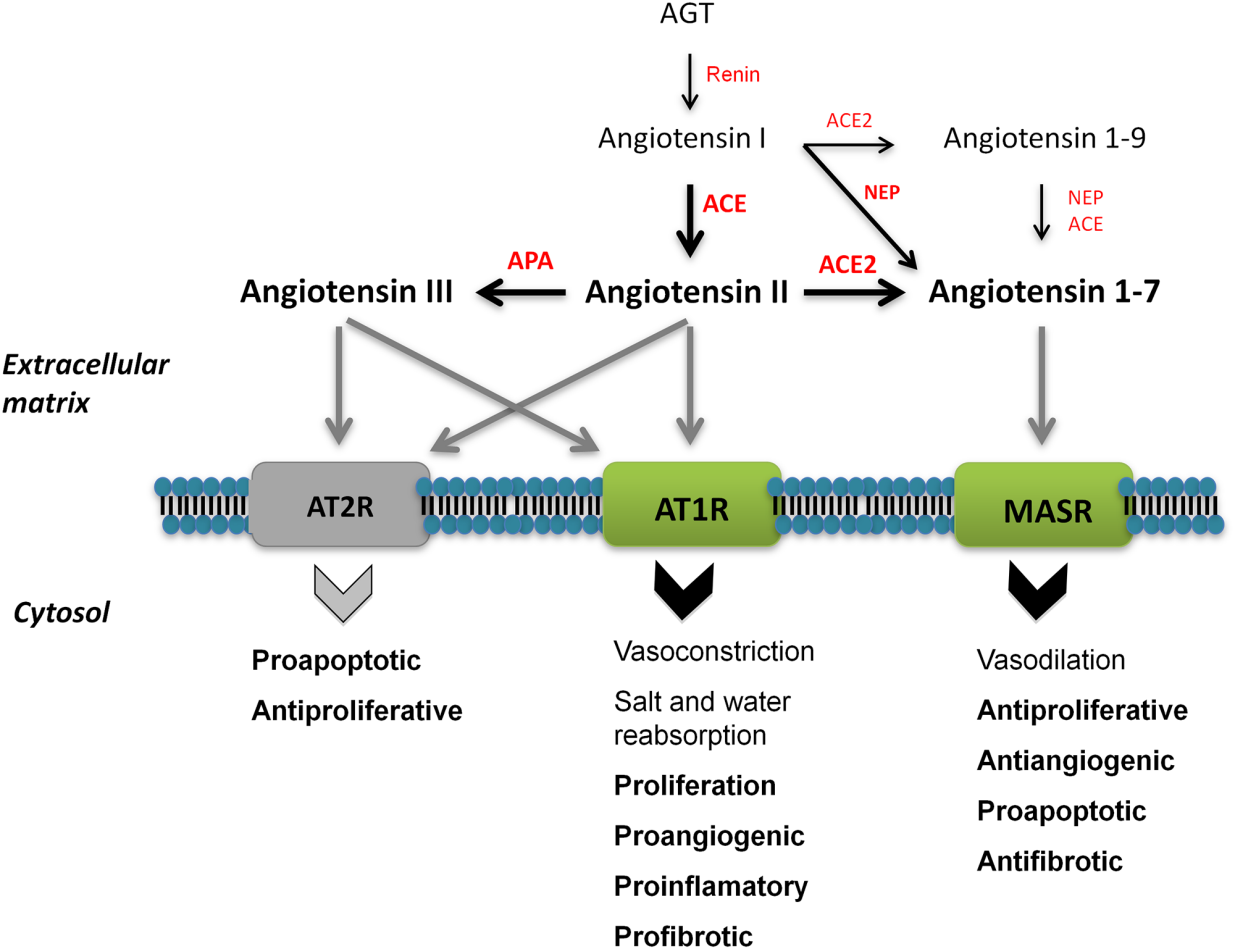
Schematic illustration of RAS and its biologic functions. Angiotensin II (Ang II), the best known bioactive peptide of RAS, is mainly generated by the catalytic action of angiotensin-converting enzyme (ACE), and binds to Angiotensin II Type 1 (AT1) and Type 2 (AT2) receptors [7–10]. Ang II is metabolized to angiotensin III (Ang III), which also binds to these receptors. Angiotensin 1–7 (Ang 1–7) is mainly produced from Ang II by ACE2, and alternatively from angiotensin I (Ang I) and angiotensin 1–9 Ang 1–9 by (NEP/CD10). Peptide transformation is symbolized by black arrows, while grey arrows show each bioactive peptide binding to their receptors. Black arrow thickness represents enzyme affinity for the substrate. Ang II has short-term cardiovascular and renal functions (normal lettering) and produces long-term local effects (represented in bold) in several tissues. These biological effects are counterbalanced by the action of Ang 1–7 [7–10].

The renin-angiotensin system (RAS) was traditionally described as an endocrine pathway that regulates the hydro-electrolytic balance and the cardiovascular function [7]. However, recently the concept of RAS has undergone an extensive revision [8,9] to cover endocrine, paracrine, autrocrine and intracrine functions regulating processes such as cell growth, proliferation and tissue repair [7,8] (see Fig 1). Imbalance in components of RAS has been associated with several chronic pathologies, including cancer [8,10,11].

The role of intrarenal RAS (iRAS) in renal carcinogenesis has been suggested by epidemiological [12–14], translational [15–18] and clinical evidences [19,20]. We have described in previous studies that the enzymatic activities of several angiotensin-regulating peptidases were significantly lower in different renal tumor subtypes when compared with the uninvolved surrounding renal tissues [21–25]. Some of these changes in enzyme activity correlated with protein and mRNA expression and were associated with tumor aggressiveness and survival [21–25].

We analyze here the immunohistochemical expression of four crucial peptidases in RAS [neutral endopeptidase/CD10 (NEP/CD10), angiotensin-converting enzyme-2 (ACE2), aminopeptidase A (APA), and angiotensin-converting enzyme (ACE)] in a retrospective series of 144 RCCs, and the fluorimetric activity of these enzymes in prospectively collected serum of 128 RCC patients. The three most common renal carcinomas (clear cell, papillary, chromophobe renal cell carcinomas) and the renal oncocytoma have been included in this study. In order to examine prognostic potential of these biomarkers we studied their relationship with survival in CCRCC cases.

## Materials and methods

### Patients

Samples and data from patients included in this study were provided by the Basque Biobank for Research OEHUN (http://www.biobancovasco.org). All patients were informed about the potential use for research of their surgically rejected tissues, and accepted this eventuality by signing a specific document approved by the Ethical and Scientific Committees of the Basque Country Health System (Osakidetza). (CEIC 2015/060, CEIC-E PI2015101 and CEIC 11–51).

Formalin fixed and paraffin embedded samples from renal tumors of 144 patients surgically treated at Cruces University Hospital between 1997 and 2001 were retrospectively selected for the study and re-reviewed under the microscope for diagnostic consistency by an experienced uropathologist. Clinical follow-up extended from RCC diagnostic data to December 31, 2015.

American Joint Committee on Cancer (AJCC) [26] and Furhman’s [27] methods were applied to assign Stage and Grade, respectively.

In addition, serum samples from 128 patients, diagnosed with RCC between 2012 and 2016 in Basurto University Hospital, were collected preoperatively. Clinical follow-up extended from RCC diagnostic data to December 31, 2016. Serum from 40 healthy volunteers with no clinical history of neoplastic diseases was used as control sample (male/female 21/19, age 55.6/59.3 years). Serum samples were from CCRCC patients with a short follow-up (average: 32.7 months). So, we used two validated scales that predict patients’ relapse probability: the UCLA Integrated Staging System (UISS) model [28], and the Mayo Clinic stage, size, grade, necrosis (SSIGN) model [29].

Table 1 summarizes the clinical and histological characteristics of patients with renal tumors.

**Table 1:** Clinical and pathological parameters of patients with renal tumors.

CCRCC Patients	Tissue samples	Serum samples
	Average (range)	Average (range)
**Age** (range)	70.69 (39–93)	61.42 (36–82)
**Sex** (Male/Female)	76/26	60/29
**Follow-up** (months)	110.3 (7–204)	32.27 (1–57)
**Survival**		
Alive	64	75
Dead of disease	38	14
**Diameter**		
≤4 cm	35	28
>4 to 7cm	33	39
>7 cm	34	22
**Grade**		
G1	21	2
G2	45	46
G3	22	30
G4	14	10
**pT**		
pT1	59	59
pT2	13	12
pT3	28	16
pT4	2	2
**PRCC Patients**		
**Age** (range)	74.79 (58–89)	54.0 (26–80)
**Sex** (Male/Female)	17/4	17/4
**ChRCC Patients**		
**Age** (range)	73.41 (58–94)	64.625 (47–74)
**Sex** (Male/Female)	11/6	7/1
**OR Patients**		
**Age** (range)	84.25 (74–93)	63.45 (40–84)
**Sex** (Male/Female)	4/0	3/7

### Tissue microarray (TMA) construction and NEP/CD10, ACE2, APA and ACE immunohistochemistry

Four tissue microarrays (TMA) were prepared for the study. Each TMA was constructed by transferring a representative part of 42 different RCC samples into a paraffin block. H&E staining was performed in order to select high tumor load (>70%) areas. Each TMA also included a set of 4 non-tumor tissue samples (2 samples from non-tumor renal tissue adjacent to two renal tumors and 2 samples of normal renal tissue from nephrectomies performed for non-tumor diseases). Non-tumor tissue controls were obtained from the same cases in the 4 TMAs.

Immunohistochemistry was performed using an automated immunostainer (AutoStainer Link 48 Dako, Glostrup, Denmark) under standard protocols for research antibodies. After deparaffination and rehydration, optimal antigen retrieval was performed in DAKO's PT Link pretreatment module and low pH target retrieval solution. Retrieved slides were incubated with antibodies against NEP/CD10 (SPM118, Santa Cruz Biotechnologies; 1:100), ACE2 (AC18Z, Santa Cruz Biotechnologies; 1:50), APA (Abcam; 1:250) and ACE (2E2), Abcam; 1:20). Characteristics of each antibody are detailed in S1 Table. EnVision Flex visualization system was used as suppliers recommend. Negative controls were processed under the same conditions as the test slides with the only difference that weren't exposed to the primary antibody.

### NEP/CD10, APA ACE and ACE2 activity measurements

Each enzyme activity was measured in triplicate by fluorometric assays in a FluoStar Optima microplate Reader (BMG Labtech, Ortenberg, Germany). Assays are based on the measurement of the fluorescence of products generated by each enzyme activity at saturating concentrations of specific substrates.

For NEP/CD10 activity measurement, method designed by Florentin et al. was employed [30]. Briefly, 30 μl of sample were incubated with 1mM of N-dansyl-D-Ala-Gly-pNO2-Phe-Gly (DAGNPG, a dansyl derivative, Sigma-Aldrich). After 30 min at 37°C generated [D]AG fluorescense intensity was measured at 560 nm with excitation at 340 nm. Thyorphan, a recognized enkephalinase inhibitor was used in a concentration of 10 μM for inhibition assays, with a 93% success.

Aminopeptidase A activity was measured by a modified version of Tobe et al's method [31]. 10 μl of serum sample were incubated with Glu-β-naphthylamide (0.227 mM) (Sigma-Aldrich), 30 min at 37°C in a final volume of 200 μl. Fluorescence intensity of generated β-naphthylamide was measured at 410 nm wavelength with excitation at 340 nm. Inhibition assays to ensure assay specificity were performed with fenantroline 1,2 mM, obtaining an inhibition of the 87% of total activity.

ACE and ACE2 activity was estimated by measuring the fluorescence intensity of the product generated by the hydrolysis of their respective specific substrates Abz-Gly-Phe(NO_2_)-Pro and Abz-Ser-Pro-NTyr-OH (Bachem). ACE was measured following the protocol designed by Santandreu et al. [32]. 50 μl of sample were incubated in a final volume of 300 μl with 0.45 mM of specific substrate for 30 min. at 37°C. For ACE2 activity measurement, a modified version of the method described by Yan ZH et al. [33] was used, incubating 30 μl of sample in 100μl of 0.5mM substrate for 2h at 37°C. Fluorescence intensity was measured in an excitation wavelength of 360 nm and emission at 410 nm in both assays. Substrate specificity was checked by inhibition assays with captopril for ACE and DX600 for ACE2. Total inhibition was observed in both cases when 10 μM and 1 μM of inhibitor were added respectively.

Blanks consisted of buffer (instead the sample) and the same reactive and were used to determine background fluorescence. Relative fluorescence was converted into picomoles of product using a standard curve constructed with increasing concentrations of *β*-naphthylamine (1.5–500 μM) [D]AG (0.625–150 μM) or Abz-Gly-OH (2.5–300 μM).

### Statistical analysis

SPSS^®^ 22.0 software (IBM, Madrid, Spain) was used to perform statistical data analysis. A Kolmogorov-Smirnov test was applied to data obtained from tissue and serum samples to determine whether the numbers followed or not a normal distribution. Based on this information, data were analyzed with non-parametric tests. We performed Spearman Rho correlation test to evaluate the correlation between RAS enzyme expression and activities, and patient age and gender. Mann-Whitney U test (Mann-U) was used to detect differences in serum enzyme activity from RCC patients and their controls, and from patients with CCRCCs with different tumor size (more or less than 7cm), grade [G1/2 (low) vs. G3/4 (high)] and stage [pT1/2 (organ confined) vs. pT3/4 (non-organ confined)]. Kruskal-Wallis test was applied to analyze serum activity differences in patients with different renal tumor subtypes and in CCRCC patients with different UISS and SSIGN scores. Chi-square (χ^2^) test was used to compare enzyme expression in different histological parameters and to analyze the relationship between categorical enzyme expression (negative or positive) and pathologic variables of CCRCC tissues.

Finally, Kaplan-Meier curves and log-rank test were performed to evaluate the association between the expression and activity of angiotensin-regulating enzymes and overall survival of CCRCC patients. Groups were created by cut-off points based on categorical enzyme expression and median of enzyme activity values. A Cox regression model (univariate and multivariate) was used to test the independent effects of clinical and pathological variables and peptidase expression/activity on survival.

A p-value lower than 0.05 (p<0.05) was considered a statistically significant result for all statistical analysis.

## Results

### Expression of RAS enzymes in different subtypes of renal tumors and in their adjacent non-tumor tissues (Fig 2)

**Fig 2:**
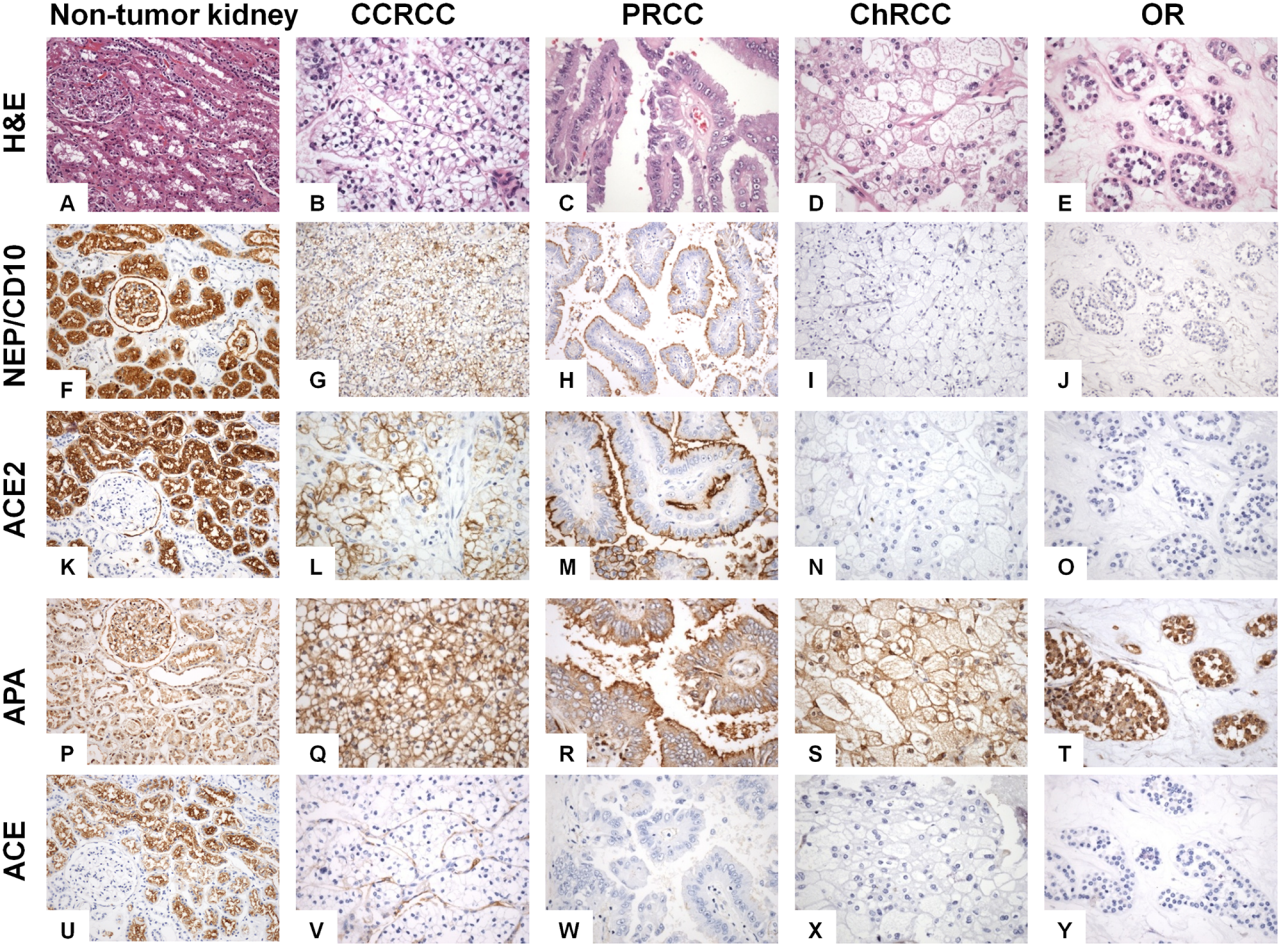
Plate showing the hematoxylin and eosin (H&E) staining of normal kidney (A), clear cell renal cell carcinoma (CCRCC) (B), papillary renal cell carcinoma (PRCC) (C), chromophobe renal cell carcinoma (ChRCC) (D) and renal oncocytoma (RO) (E) and immunohistochemical pattern displayed for four peptidases (NEP/CD10, ACE2, APA and ACE) (test performed in formalin-fixed and paraffin-embedded material). NEP/CD10 immunostaining was positive in proximal convoluted tubules of the normal kidney (F), as well as in about half CCRCC (membranous) (G) and PRCC (apical membranous) studied samples (H). By contrast, it was negative in ChRCC (I) and RO (J). ACE2 immunostaining was positive also in proximal convoluted tubules of the normal kidney (K), as well as in CCRCC (membranous) (L) and PRCC (apical membranous) (M). By contrast, it was negative in almost 60% of ChRCC (N) and in all RO (O). APA immunostaining was diffusely positive in normal kidney (tubules and glomeruli) (P), as well as in CCRCC (membranous) (Q), PRCC (membranous) (R), ChRCC (membranous and cytoplasmic) (S) and RO (cytoplasmic) (T). ACE immunostaining was positive in proximal convoluted tubules of the normal kidney (U). All CCRCC (V), PRCC (W), ChRCC (X) and RO (Y) were negative. Only neoplastic blood capillaries were positive in CCRCC (V). (Original magnification, x20 for uninvolved renal tissues, and x400 for tumors).

NEP/CD10 immunostaining was positive in proximal convoluted tubules of the normal kidney (Fig 2F), as well as in almost half of studied CCRCC (membranous) (Fig 2G) and PRCC (apical membranous) samples (Fig 2H). By contrast, it was negative in ChRCC (Fig 2I) and RO (Fig 2J). ACE2 immunostaining was positive also in proximal convoluted tubules of the normal kidney (Fig 2K), as well as in CCRCC (membranous) (Fig 2L) and PRCC (apical membranous) (Fig 2M). On the contrary, it was negative in almost 60% of ChRCC (Fig 2N) and all RO (Fig 2O). APA immunostaining was diffusely positive in normal kidney (tubules and glomeruli) (Fig 2P), as well as in CCRCC (membranous) (Fig 2Q), PRCC (membranous) (Fig 2R), ChRCC (membranous and cytoplasmic) (Fig 2S) and RO (cytoplasmic) (Fig 2T). ACE immunostaining was positive in proximal convoluted tubules of the normal kidney (Fig 2U). Tumor cells from all CCRCC (Fig 2V), PRCC (Fig 2W), ChRCC (Fig 2X) and RO (Fig 2Y) were negative. However, in CCRCC, neoplastic blood capillaries were positive (Fig 2V).

Expression of both NEP/CD10 and ACE2 were variable in RCC histotypes (chi-square χ^2^ test p = 0.006 for both markers) (Table 2). Half of the CCRCC (45.6%) and PRCC (42.9%) expressed NEP/CD10. In contrast, very few ChRCC (5.9%) and no RO cases expressed it. Differences were significant between CCRCC and ChRCC (chi-square χ^2^ test p = 0.002), PRCC and RO (p = 0.01), and almost between CCRCC and RO (p = 0.07).

**Table 2:** Expression of RAS enzymes on RCC according to histological subtype. NEP/CD10, ACE2 and APA expression was studied in renal carcinoma cells while ACE expression was in intratumor blood vessels.

Histologic subtype	n	NEP/CD10			ACE2			APA			n	ACE		
		Neg (%)	Pos (%)	P value	Neg (%)	Pos (%)	P value	Neg (%)	Pos (%)	P value		Neg (%)	Pos (%)	P value
**CCRCC**	102	54.4	45.6	**0.006**	30.1	69.9	**0.006**	20.6	79.4	0.334	99	57.4	42.6	**0.002**
**PRCC**	21	57.1	42.9		38.1	61.9		14.3	85.7		19	94.7	5.3	
**ChRCC**	17	94.1	5.9		58.8	41.2		17.6	82.4		17	88.2	11.8	
**RO**	4	100.0	0.0		100.0	0.0		25.0	75.0		3	100.0	0.0	

Similarly, roughly two thirds of CCRCCs and PRCCs expressed ACE2, whereas less than a half of ChRCC and no RO did it (Table 2). Statistically significant differences were founded when CCRCC was compared with ChRCC (chi-square χ^2^ test p = 0.021) and RO (p = 0.003), and between PRCC and RO (p = 0.023).

APA expression was detected in more than 75% renal tumors studied and no significant differences in the expression of APA were observed among histological subtypes. ACE, in contrast, was expressed in almost a half of CCRCCs (Table 2), whereas the other histological subtypes were almost all negative in PRCC (chi-square χ^2^ test p = 0.002) and ChRCC (p = 0.017), or negative in RO (p = 0.1).

#### RAS enzymes expression in CCRCC patients according to clinical and pathologic variables

There was not any correlation between enzyme expression and patients’ sex and age (Spearman Rho test p>0.05 for all cases).

**Fig 3:**
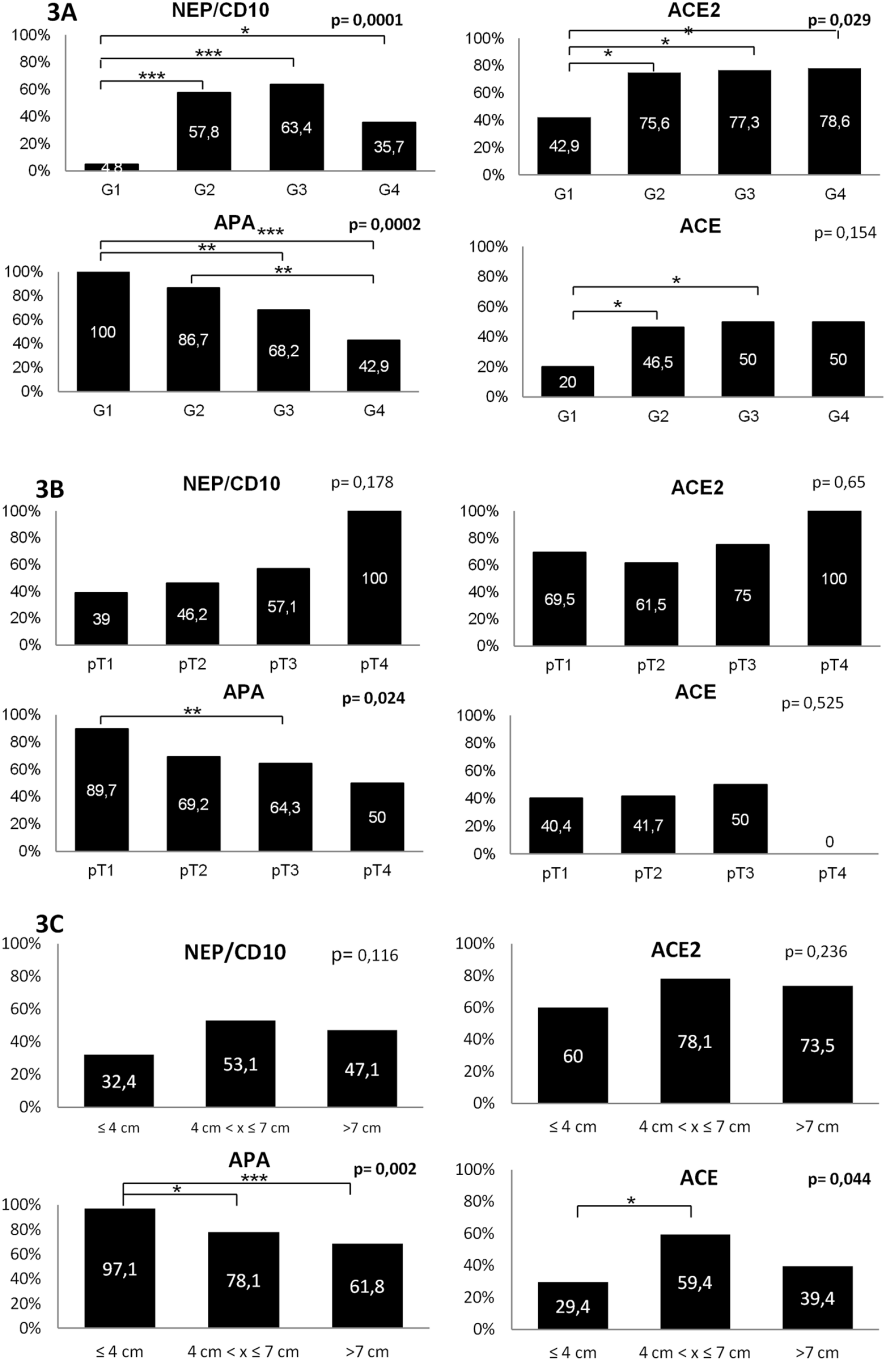
Expression of RAS enzymes in CCRCC according to fuhrmans' grade (A), AJCC tumor stage (B) and tumor size (C). Percentage of positive (black) samples for each enzyme are represented in figure bars. Histological grade (G1-G4), pathological stage (pT1-pT4) and tumor size (≤4cm - 4cm<x≤7cm—>7cm) intergroup differences were evaluated using χ^2^ test. Statistically significant correlations are highlighted in bold. Statistically significant differences between two specific groups were also analyzed using χ^2^ test((*)p<0.05; (**)p<0.01; (***)p<0.005).

In contrast, the expression of RAS enzymes correlated significantly with pathological parameters of tumor aggressiveness such as histologic grade, stage and size (Fig 3).

Thus, when data were stratified by histologic Grade (Fig 3A), significant differences were observed for NEP/CD10 (chi-square χ2 test p = 0.0001), ACE2 (chi-square χ2 test p = 0.029) and APA (chi-square χ2 test p = 0.0002). When comparisons were performed between two groups, NEP/CD10 expression was significantly lower in G1 than in the rest tumor grades (G1 vs G2 p = 0.000062; G1 vs G3 p = 0.000051; G1 vs G4 p = 0.017). ACE2 showed similar results (G1 vs G2 p = 0.011; G1 vs G3 p = 0.021; G1 vs G4 p = 0.036). On the contrary, APA expression in tumor cells decreased while tumor grades increased (G1 vs G3 p = 0.006; G1 vs G4 p = 0.00011; G2 vs G4 p = 0.001). We also observed lower ACE expression in blood vessels from G1 than in higher grade CCRCCs when data were compared between two groups (G1 vs G2 p = 0.044; G1 vs G3 p = 0.043).

With respect to tumor stage (Fig 3B), APA expression was decreased in higher stage CCRCCs (chi-square χ2 test p = 0.024). This difference was significant between pT1 and pT3 tumors (p = 0.005), and almost significant between pT1 and pT2 (p = 0.06), and pT1 and pT4 (p = 0.04). We did not found any significant result for NEP/CD10 (p = 0.178), ACE2 (p = 0.65) and ACE (p = 0.525).

Regarding tumor size (Fig 3C), results were stratified in three groups following previously reported classifications [28, 34]: tumors with 4cm or smaller, 4 to 7cm tumors, and tumors larger than 7cm. APA and ACE expression showed statistically significant differences (chi-square χ2 test p = 0.001 and p = 0.044, respectively). APA expression was higher in CCRCCs smaller than 4cm with respect to tumors between 4-7cm (p = 0.019) and larger than 7cm (p = 0.0003). Inversely, ACE expression was lower in CCRCCs smaller than 4cm with respect to tumors between 4-7cm (p = 0.014). NEP/CD10 (p = 0.12) and ACE2 (p = 0.24) did not throw significant results.

#### Tissue RAS enzyme expression associated with overall survival in CCRCC patients

**Fig 4:**
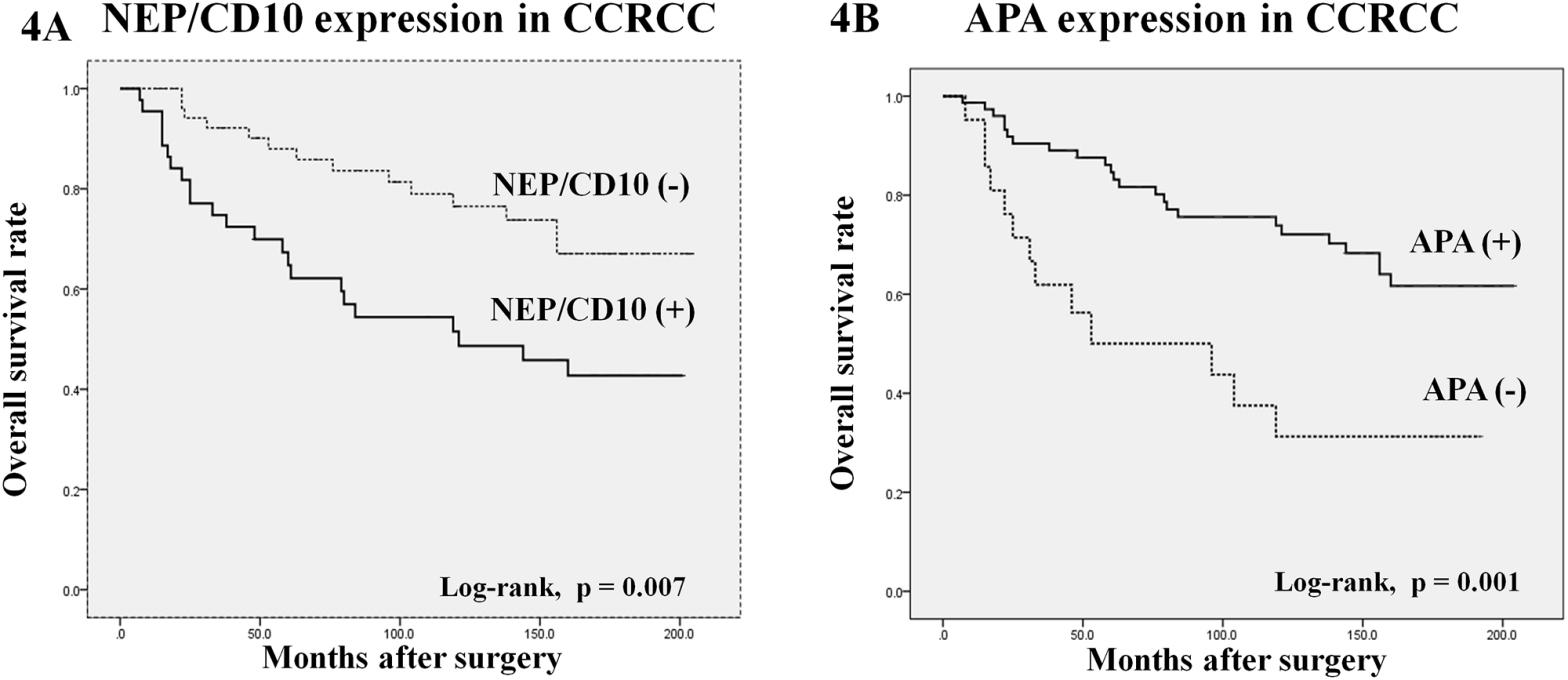
Kaplan-Meier survival curves for NEP/CD10 (A), and APA (C) expression in clear cell renal cell carcinomas. 15-year overall survival showed significant differences between protein positive and negative cases (log-rank test p<0.05).

Expression of NEP/CD10 in tumors was associated with worse survival (log-rank p = 0.007) (Fig 4A) (Table 3). Inversely, APA expression was correlated with better survival of CCRCC patients (log-rank p = 0.001). ACE2 and ACE expression did not correlate with patient survival. (Table 3).

**Table 3:** Log-rank and cut-off values for angiotensin-regulating enzymes predicting overall survival of CCRCC patients. Statistically significant are highlighted in bold.

**IHC Expression**	**Cut-off value**	**Log-rank p =**
NEP/CD10	Pos/Neg	**0,007**
ACE2	Pos/Neg	0,177
APA	Pos/Neg	**0.001**
ACE	Pos/Neg	0,219
**Enzyme activity**	**Cut-off value**	**Log-rank p =**
NEP/CD10	7350	**0,007**
ACE2	730	0,518
APA	2350	0,52
ACE	650	0,206

By univariate analysis, overall survival correlated with tumor diameter, grade, pathologic stage, and NEP and APA expression. These variables were included in the Cox regression multivariate analysis and results indicated that tumor stage (pT), NEP and almost APA expression were independent prognostic factors predicting CCRCC patients’ survival (Table 4).

**Table 4:** Univariate and multivariate analysis (Cox regression model) of clinicopathological variables and NEP/CD10 and APA expression predicting overall survival of patients with CCRCC. Statistically significant correlations are highlighted in bold.

	Univariate analysis		Multivariate analysis for NEP/CD10		Multivariate analysis for APA	
Variables	p value	OR	p value	OR	p value	OR
**Sex**	0.911	1.042	-	-	-	-
**Age**	0.064	1.026	-	-	-	-
**Diameter**	**0.00001**	2.83	0.327	1.335	0.863	1.054
≤4 cm						
>4 to 7cm						
>7 cm						
**Grade**	**0.000019**	2.047	0.421	1.194	0.533	1.144
G1						
G2						
G3						
G4						
**Stage**	**5.24·10**^**−10**^	3.395	**0.001**	2.534	**0.00014**	1.054
pT1						
pT2						
pT3						
pT4						
**NEP/CD10 expression**	**0.003**	2.886	**0.047**	2.001	-	-
Positive						
Negative						
**APA expression**	**0.001**	0.295	-	-	0.063	0.473
Positive						
Negative						

### Serum activity of RAS enzymes in patients with renal tumors with respect to healthy subjects

Serum activity of NEP/CD10, ACE2 and APA and was higher in patients with renal tumors than in healthy subjects. This result was statistically significant for NEP/CD10 only in CCRCC patients; in all of tumor subtypes for ACE2; and in patients with CCRCC, PRCC and RO for APA (Table 5). ACE activity was lower in ChRCC and RO than in controls, however, this difference did not reach statistical significance.

**Table 5:** Serum activity of RAS enzymes on renal tumor patients and healthy controls.

	Control	CCRCC	PRCC	ChRCC	RO
**NEP/CD10**	7183,58 ± 605,6	7453,01 ± 733,7*	7028,14 ± 1085,4	7402,25 ± 519,7	7516,55 ± 422,5
**APA**	2087,6 ± 401,6	2486,55 ± 656,7***	2562,95 ± 596,0**	2408,75 ± 573,1	2528,45 ± 633,1*
**ACE2**	549,00±206,15	767,966±304,45***	875,667±520,17***	666,00±160,36*	662,9±112,77**
**ACE**	619,4±238,8	679,18±192,4	659,24±195,4	513,25±124,7	562,64±147,3

NEP/CD10 activity was higher in patients with CCRCC than in healthy subjects, ACE2 activity was higher in all histological subtypes and APA was higher in CCRCC, PRCC and RO patients. Values are means ± SE of units of peptidase per liter of plasma (UP/L). Statistically significant results are highlighted in bold (Mann-U p<0.05 *, p<0.01 **, p<0.001 ***).

### Activity of angiotensin-regulating enzymes in serum of CCRCC patients according to clinical and pathologic variables

There was no correlation between the activity of serum RAS enzymes and sex and age of CCRCC patients (Spearman Rho test p>0.05 for all cases). We neither found any significant result when data were stratified by tumor size (Kruskal-Wallis p>0.05).

Serum activity of RAS enzymes correlated with pathological parameters of tumor aggressiveness. Thus, lower ACE activity was observed in serum from patients with high grades (G1/G2 717.38 ± 187.94 vs G3/G4 634,5 ± 192,592; Mann-U p = 0.029) and metastatic CCRCC (local disease 706,1 ± 192,81 vs metastatic disease 565.18 ± 147.05; Mann-U p = 0.009). NEP/CD10 activity showed a similar trend, although this result did not reach statistical significance. Activity of the other two enzymes didn't show statistically significant results when stratified with these variables.

**Fig 5:**
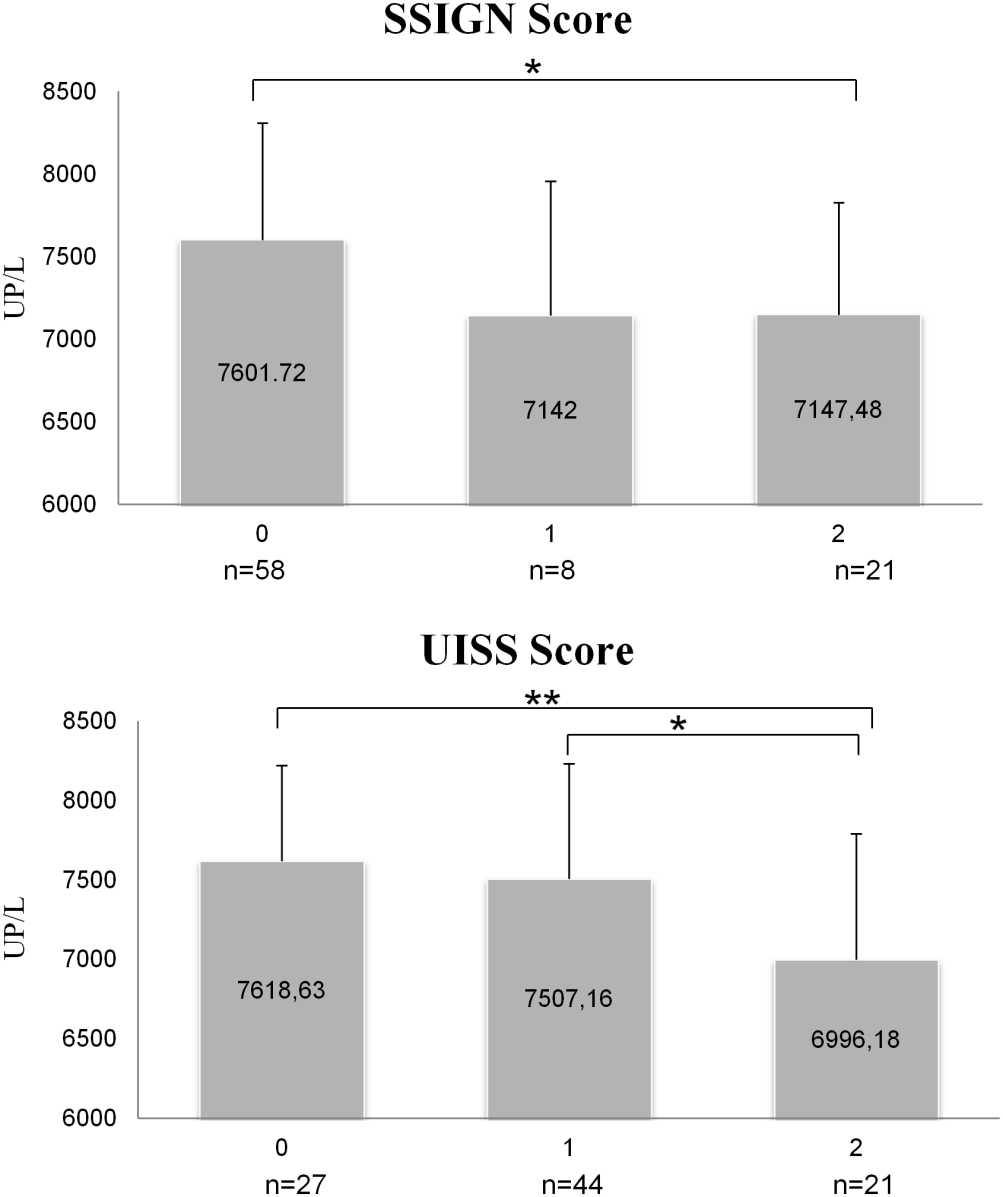
Serum activity of NEP/CD10 in CCRCC patients according to UISS and SSIGN scores. Kruskal-Wallis test showed statistically significant differences both for NEP/CD10 activity stratified by SSIGN (p = 0,03) and UISS (p = 0.027). By SSIGN score, differences were founded between low progression patient group and high progression patient group (Mann-U, p = 0.014). By UISS score, differences were detected between high mortality risk group when compared with low (Mann-U, p = 0.008) and intermediate (Mann-U, p = 0.022) risk groups. Values are means ± SE of units of peptidase per liter of plasma (UP/L).

When NEP/CD10 activity was stratified by SSIGN model, which predicts progression of CCRCC after radical nephrectomy [29], lower activity was measured in the group of patients with high (= 2) or intermediate (= 1) progression risk when compared with lower (= 0) progression risk group (Fig 5A) (Kruskal-Wallis test p = 0.03). And stratified by UISS score, NEP/CD10 activity in serum from patients with high mortality risk (= 2) was also lower than in patients with low (= 0) and intermediate (= 1) mortality risk (Fig 5B) (Kruskal-Wallis test p = 0.027). Activity of the other 3 enzymes didn't show statistically significant results when stratified with these predictive models.

### RAS enzyme activity according to overall survival of CCRCC patients

**Fig 6:**
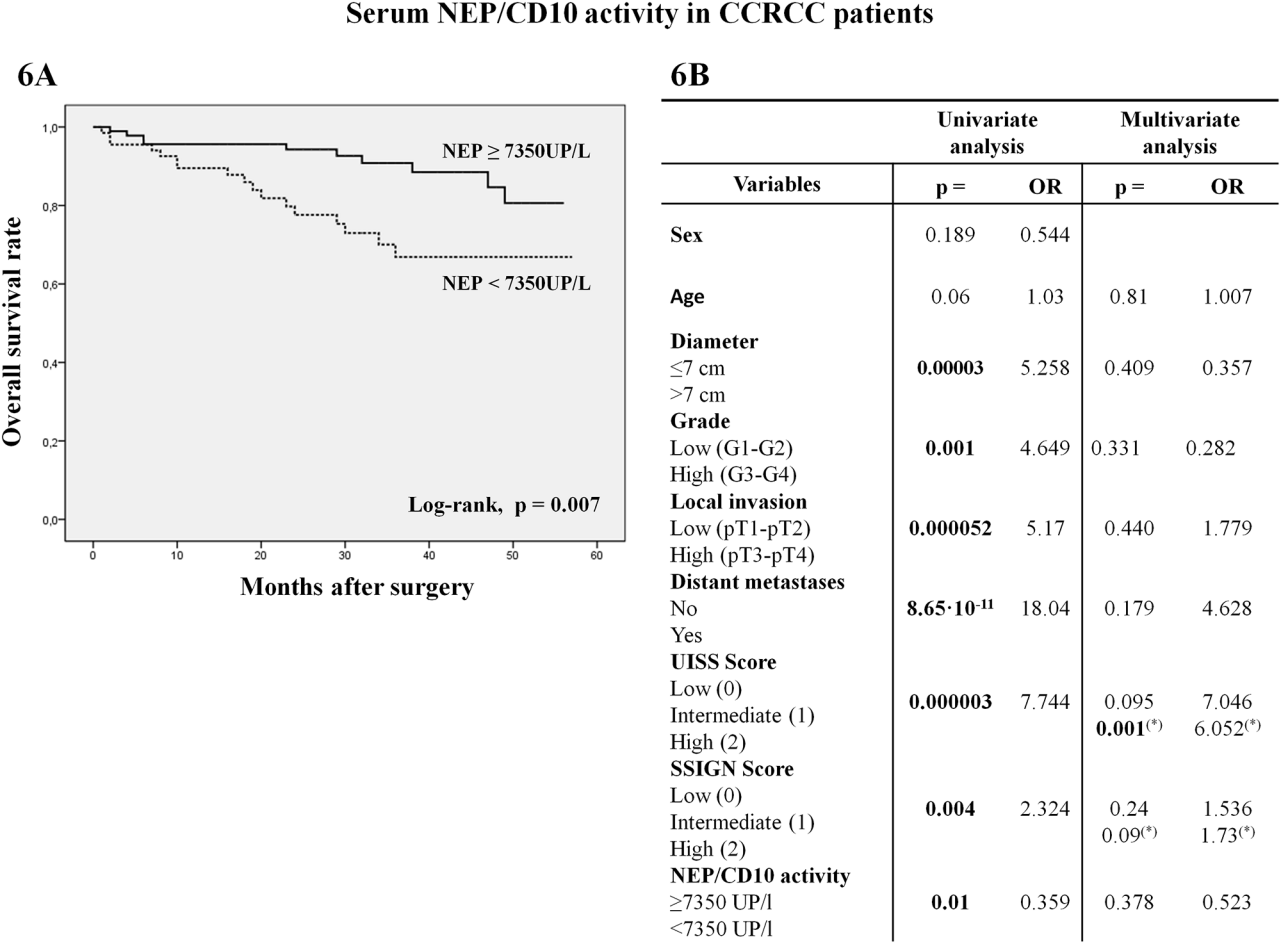
Kaplan-Meier survival curves for NEP/CD10 activity in serum from CCRCC patients. **(A)** Overall survival from patients was worse when enzyme activity was lower than 7350 UP/L. **(B)** Univariate analysis (Cox regression model) of clinicopathological variables showing significant results for serum NEP/CD10 activity. By multivariate analysis (backward conditional procedure), UISS and SSIGN scores remained as the most significant variables predicting patients’ survival. ^(*)^Probability (p) and odds ratio (OR) in the final step by the backward conditional method.

The analysis of NEP/CD10 activity in serum samples displayed opposite results to those obtained in tumor tissue. Thus, when serum NEP/CD10 activity was lower than 7350 UP/L, the overall survival was significantly worse according to Kaplan-Meier curves (Fig 6A) (log-rank, p = 0,007).

Univariate analysis showed that tumor diameter, grade, pT, distant metastases, UISS and SSIGN scores, NEP/CD10 activity and almost patients’ age are associated with overall survival. These variables were included in the Cox multivariate analysis and a stepwise selection procedure (backward conditional method) was used to select the optimal predictive model. This analysis showed that UISS (p = 0.001) and SSIGN (p = 0.09) scores were the most relevant variables predicting CCRCC patients’ survival, although SSIGN score did not achieved statistical significance.

## Discussion

A wide variety of tissues and organs express all the components of the RAS, where they have important homeostatic functions in cell differentiation and proliferation. In contrast their deregulation may contribute to the development and progression of proliferative disorders [8,11]. The discovery of an intrarenal RAS (iRAS) has been crucial to understanding the renoprotective effect of ACE inhibitors (ACEi) and Ang II receptor blockers (ARB) in non neoplastic kidney diseases with fibrotic and proliferative characteristics such as diabetic nephropathy [35]. Additionally, it has opened new perspectives in kidney carcinogenetic processes and in the potential usefulness of these drugs in renal cancer [13–18,20].

Angiogenesis is a key factor in RCC development [36]. Increasing evidence demonstrates that imbalances in RAS favoring the ACE–Ang II–AT1 receptor axis play a critical role in vascular endothelial growth factor (VEGF)-dependent angiogenesis [8,10,11]. It has been described that the expression of AT1 and AT2 receptors correlates with tumor grade and with worse survival in CCRCC patients [16]. Recent studies in xenograft models of human RCC have showed that the blockade of this axis with ACEi and ARB decreases tumor diameter, vascularization and metastatic capacity, and enhances the antiangiogenic effect of sunitinib [15,17]. Moreover, retrospective analyses [12,14] and a clinical trial [20] have demonstrated that the use of RAS inhibitors improves survival in metastatic CCRCC patients receiving VEGF-targeted therapy.

We reported previously that ACE activity was higher in high grade CCRCC tissue homogenates than in low grade ones [24]. Interestingly, ACE was not expressed in tumor cells but rather in endothelial cells, suggesting that the source of ACE activity was the tumor vasculature. However, the immunohistochemical analysis of this study was performed in few prospectively collected cases [24]. In the present study we have analyzed the expression of this key enzyme in the generation of angiotensin II [37] in a retrospective series of 144 renal neoplasms. The immunohistochemical analysis has corroborated the exclusive location of this peptidase in tumor vessels and has shown a higher immunostaining in CCRCC, the most aggressive renal tumor subtype [3] and, specifically, in high grade cases.

On the other hand, we also have analyzed the expression of APA, another key peptidase of RAS that converts Ang II to Ang III [38]. Inversely to that observed with ACE, this enzyme was expressed in CCRCC cells but not in blood vessels. Moreover, APA expression was lower in larger and higher grade tumors, and the loss of expression was correlated with worse survival. A similar decreasing pattern of APA expression was reported in gynecologic malignancies and indicated that this enzyme may have a tumor suppressor role by degrading Ang II [39,40].

Taking these results into account, we hypothesize that the higher ACE expression in tumor vessels and the lower APA expression in CCRCC tumor cells could lead to an accumulation of Ang II in tumor microenvironment, thereby stimulating angiogenesis and promoting tumor growth and invasiveness. Further studies are needed to examine this hypothesis and clarify the underlying mechanism of action of RAS inhibitors in the enhancement of the antiangiogenic effects of VEGF-targeted therapies [12,14,15,17,20].

Ang 1–7 is a bioactive peptide of RAS mainly generated from Ang II by ACE2, and alternatively from Ang I and Ang 1–9 by NEP/CD10 [37]. It is generally accepted that Ang 1–7 has counter-regulatory effects on many of the events induced by Ang II [7]. Thus, in non-neoplastic renal diseases this peptide inhibits proinflammatory and profibrotic processes that lead to renal damage and ACEi and ARBs have local tissue effects restoring imbalances between Ang II/AT1 and Ang 1-7/Mas receptor axis [41,42]. Furthermore, it has been described in several solid tumors that Ang 1–7 inhibits angiogenesis and tumor progression, and suggested that the balance between two axis may be important in determining if tumors gain an angiogenic and invasive phenotype [8,10,11,19,43].

In this context, it might be expected that Ang 1–7 has an antitumor effect in renal tumors. However, a recent study demonstrated that this peptide increases metastatic potential of RCC cells through its Mas receptor and AKT signaling pathways [18]. Consistent with these findings, we observed that ACE2 and NEP/CD10 expression was higher in proximal nephron-derived carcinomas (CCRCC and PRCC), which are more aggressive than distal-nephron derived carcinomas (ChRCC) [3]. Moreover, both peptidases were highly expressed in higher grade CCRCCs and NEP/CD10 was independently correlated with worse patients’ survival. Taken together, these results suggest a different role for the Ang 1–7 axis in renal cancer and, therefore, that RAS imbalances in carcinogenetic processes are tumor-specific [8,10,11,18].

The best known function of peptidases is the conversion of bioactive peptides [44]. However, these enzymes can also regulate cell growth, differentiation and signal transduction of many cell systems by degrading extracellular matrix molecules and directly participating in the intracellular signaling as receptors [44–46]. The present study and other previous [21–25] have described that angiotensin-regulating peptidases show different expression and activity patterns in RCCs when compared with the non-tumor surrounding tissue or when stratified by tumor aggressiveness. For example, ACE, ACE2 and NEP/CD10 activity was markedly decreased in renal tumors with respect to normal tissues [21–25]. However, ACE activity [24] and expression, and NEP/CD10 and ACE2 expression was higher in high grade CCRCCs. Therefore, it should be taken into account that the role of these peptidases in the different stages of renal cancer could be due to the sum of its different biological actions or, rather, to its net result [44–46].

Angiotensin-regulating peptidases can be secreted to the extracellular space and appear in serum [44]. The comparative studies of peptidase profiles among cancer patients and control subjects have yielded significant results in several studies [47–49]. In our study, NEP/CD10, ACE2 and APA activity was higher in renal cancer patients than in healthy controls. This result agrees with those described in pancreatic and colorectal cancer [48,49] but it is opposite to that demonstrated in breast cancer [47], which suggests that tumor-specific changes of angiotensin-regulating enzymes occur also in serum of cancer patients.

Correlation among serum values of RAS enzymes and classic histopathological parameters for tumor aggressiveness and survival have pointed to these proteins as useful biomarkers for helping in screening, diagnosis, staging, prognosis and monitoring cancer therapy [47–52]. Our results support this idea. On the one hand, ACE activity was lower in serum of high grade and metastatic CCRCC patients. On the other, NEP/CD10 activity was lower in serum of CCRCC patients with high UISS and SSIGN scores. These scales predict patients’ relapse probability [28,29] and, interestingly, Kaplan-Meier curves performed with a median follow-up of 32 months corroborated this result. Further studies with higher number of patients with longer follow-ups would be advisable to confirm the prognostic significance of these data.

The origin of these circulating peptidases in neoplastic diseases is still controversial. The main hypothesis places the origin in tumor microenvironment. However, other sources (such as immune system, liver and spleen) have been also suggested [44, 52] since serum peptidase levels and activity don’t usually reflect parallelisms which occur in tumor tissues [44,47,48,52,53]. For example, in this and in a previous work [24], we observed that ACE expression and activity was higher in more aggressive CCRCCs, while serum ACE activity in CCRCC patients was lower. A similar negative correlation was observed with NEP/CD10. Besides, serum APA and ACE2 activity was similar in CCRCC patients with different prognosis while protein expression was significantly altered in aggressive tumors from patients with bad prognosis. This phenomenon needs further clarification because the source of serum peptidases seems critical to validate these enzymes as reliable markers of diagnosis and prognosis in patients with cancer [44,52,53,54].

In conclusion, the present study shows that ACE in tumor vessels and APA, ACE2 and NEP/CD10 in tumor cells are differentially expressed in different renal tumor subtypes, and that the presence/abscense of these enzymes is significantly associated with tumor aggressiveness and poor outcome of patients with CCRCC. Furthermore, serum activity of NEP/CD10, ACE2 and APA is higher in RCC patients than in healthy subjects and ACE and NEP/CD10 activity is negatively correlated with CCRCC patients’ prognosis. These results favor the possibility of a metabolic imbalance of iRAS and a role of angiotensin-regulating peptidases in renal neoplastic diseases. A better understanding of the pathophysiological role of these enzymes in these proliferative disorders will be helpful for designing effective diagnostic, prognostic and therapeutic tools for renal neoplasms.

## Supporting information

S1 Table Summary table of the antibodies employed for angiotensin converting enzymes immunohistochemical detection. (DOCX)

## Abbreviations

*ACE*: *angiotensin converting enzyme*
*ACE2*: *angiotensin converting enzyme 2*
*Ang*: *angiotensin*
*APA*: *aminopeptidase A/APA*
*CCRCC*: *clear cell renal cell carcinoma*
*ChRCC*: *chromophobe cell renal cell carcinoma*
*iRAS*: *intrarenal RAS*
*NEP/CD10*: *neutral endopeptidase/CD10*
*PRCC*: *papillary renal cell carcinoma*
*RAS*: *renin-angiotensin system*
*RCC*: *renal cell carcinoma*
*RO*: *renal oncocytoma*
