# Supplementary material for: Expression and activity of angiotensin-regulating enzymes is associated with prognostic outcome in clear cell renal cell carcinoma patients
Source: PLoS One. 2017 Aug 15;12(8):e0181711. doi: 10.1371/journal.pone.0181711 (PMC5557356; doi:10.1371/journal.pone.0181711)
Supplement: S1 Table — (DOCX) [file pone.0181711.s001.docx]

| **Antibody** | **Commercial** | **Description** | **Aplications** | **Reactivity** | **Inmunogen** | |
| --- | --- | --- | --- | --- | --- | --- |
| **Anti CD10 (SPM118) sc-52994** | Santa Cruz | Mouse monoclonal | WB,IF,IP, IHC(P) | H,R | Raised against extracellular domain of human CD10 |  |
| **Anti ACE2 (AC18Z) sc-73669** | Santa Cruz | Mouse monoclonal | WB,IF,IHC(P) | H | Raised against Aa 18-740 of hACE2 | |
| **Anti Aminopeptidase A ab109775** | Abcam | Rabbit polyclonal | WB, IHC(P) | H | Recombinant full length protein corresponding to Human Aminopeptidase A. | |
| **Anti ACE ab11734 (2E2)** | Abcam | Mouse monoclonal | WB, IHC(P), ELISA | H,M,R,Rab,Cow,Pig… | This clone recognises denatured ACE, binding to an epitope in the C domain, and has been reported to be active in WB | |
